# Effect of Cognitive Training on Daily Function in Older People without Major Neurocognitive Disorder: A Systematic Review

**DOI:** 10.3390/geriatrics4030044

**Published:** 2019-07-18

**Authors:** Brian J.Y. Fan, Roger Y.M. Wong

**Affiliations:** 1 Department of Medicine, University of British Columbia, Vancouver, BC V5Z 1M9, Canada; 2 Division of Geriatric Medicine, University of British Columbia; Vancouver, BC V5Z 1M9, Canada

**Keywords:** cognitive therapy, aging, instrumental activities of daily living, daily functioning

## Abstract

There is increasing interest in the effect of non-pharmacological treatments on preserving cognition and function in older adults without major neurocognitive disorder (dementia). However, its effect on everyday function in terms of instrumental activities of daily living (IADL) is unclear. We conducted a systematic review to examine whether cognitive training, independent of other interventions, can improve IADL function in older adults without major neurocognitive disorder. We searched multiple databases including MEDLINE, EMBASE, and PSYCINFO and found thirteen studies that met our inclusion criteria with 7130 participants in total. Six out of thirteen studies reported a significant change on validated IADL assessment. On subgroup analysis, five studies included older adults with normal cognition and one included mild cognitive impairment (MCI). Eleven out of twelve studies showed improvement in measures of cognition. None of the studies described changes in the ability to live independently. While variation in study protocol, outcome measurement, and effect size reporting precluded further inferential statistical analysis, our review found a sizable number of studies showing improvement in IADL. Cognitive training may have some benefit in improving IADL function in older adults without major neurocognitive disorder. Future long-term studies focusing on maintained IADL function and preserved independence are needed.

## 1. Introduction

The prevalence of older adults with cognitive impairment is rising globally [1]. This is associated with rising health care costs and loss of independence [2]. Strategies for major neurocognitive disorder (dementia) prevention are key to fostering the overall health of an increasing elderly population [3]. Multiple strategies have been suggested and include vascular risk factor modification by reducing the burden of cerebrovascular disease, physical activity, and increasing access to early education [4]. Cognitive training exercises have been suggested as another tool to maintain or improve cognition and promote healthy aging.

Cognitive training exercises are a group of protocolized tasks that target one or more domains of cognition [5]. They can be performed in groups or with individuals and can be computerized or face-to-face. There have been a number of studies in the last ten years investigating the role of cognitive training and its short- and long-term effects on cognition [6,7,8,9]. As highlighted in a review by Kelly et al., multiple studies have found improvements with memory, processing speed, and visuospatial processing [10]. Other recent systematic reviews, such as from Mewborn et al., have found modest, but significant, improvements in overall cognition that may be maintained in the long-term [11].

However, the effect of cognitive training exercises on daily functioning remains unclear, with recent Cochrane reviews finding only low to moderate quality studies. [12,13]. Previous studies have shown that multiple cognitive domains are required to perform complicated tasks of daily living. Executive functioning, a complex set of cognitive functions that enable planning, organizing and effective action, has been identified as a key domain [14]. However, the role of cognitive training in improving daily functioning has not been thoroughly explored.

The purpose of our review is to summarize the current literature regarding the effects of cognitive training in older adults with normal cognition and those with mild cognitive impairment. To that effect, we have found a sizable number of studies that report on the positive effects of cognitive training on daily functioning in terms of assessments of instrumental activities of daily living.

## 2. Materials and Methods

### 2.1. Study Design

We included studies that explicitly investigated the effect of cognitive training on community dwelling adults, average age greater than 65, with either no known cognitive impairment or mild cognitive impairment. We excluded studies that included co-interventions such as aerobic physical activity in their intervention arm and those that included individuals with dementia. Studies needed to have at least 10 participants in each arm and the intervention needed to be longer than 6 sessions and greater than 3 weeks. Risk of bias was performed using the PEDro criteria [15]. Our primary outcome was change in daily functioning in terms of instrumental activities of daily living. Our secondary outcomes included change in independence and measures of cognition. This study was registered on PROSPERO (CRD42018108108).

### 2.2. Search Strategy, Data Collection, Study Selection

**Figure 1:**
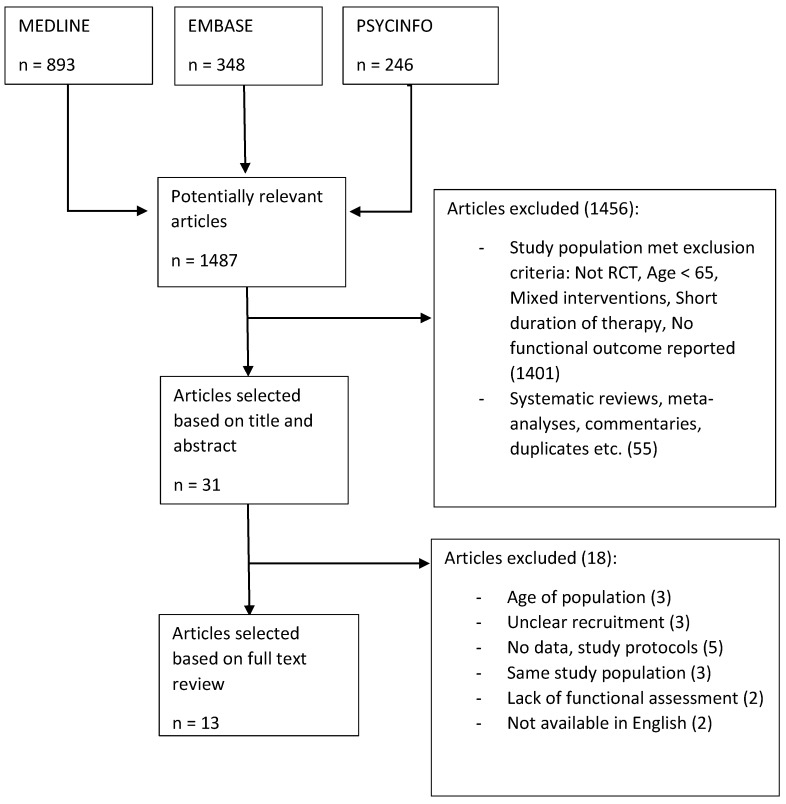
Selection criteria.

We searched multiple databases including MEDLINE, EMBASE, and PSYCINFO for randomized control trials that met our inclusion criteria published up until August 2018, following PRISMA (Preferred Reporting Items for Systematic Reviews and Meta-Analyses) guidelines. Search terms included “cognitive training”, “cognitive therapy”, “brain training’, “geriatric”, “aged”, “daily functioning”, “instrumental activities of daily living”, and “activities of daily living”. This was supplemented by screening references of previous reviews. Titles and abstracts were then screened and those that did not meet inclusion criteria were excluded. Full text analysis was then performed on the remaining studies by one reviewer. When there was uncertainty about a study, a second independent reviewer was involved and made a final decision. Our study selection process can be summarized in Figure 1.

## 3. Results

Thirteen studies met the inclusion criteria with 7130 study participants in total. Of these, eight included patients with normal cognition, four included patients with mild cognitive impairment, and one include both. Study characteristics are listed in Table 1 and Table 2. There was large variation among the studies in terms of number of study participants, follow up, and training protocol in both groups. Given the variability in the reporting of effect sizes, further inferential statistical analysis could not be performed as per the biostatistician’s advice. Our data is therefore presented in a narrative manner.

### 3.1. Normal Cognition

Eight studies included participants with normal cognition. Studies ranged from around 60 participants (Rizkalla, 2015) to 2912 (Corbett et al., 2015). Follow up also ranged from 2.5 months (Edwards et al., 2005; Giuli et al., 2016; Chen et al., 2018) to 26 months (McDougall et al., 2010). Seven of these studies were performed in ambulatory clinic and consisted of small group or computerized tasks while one study (Corbett et al., 2015) consisted of solely computerized online based tasks.

The results of these studies are summarized in Table 3. Overall, five of the eight studies reported improvement in measures of instrumental activities of daily living (IADL) (Ball et al., 2002; Edwards et al., 2005; Corbett et al., 2015; Rizkalla, 2015; Chen et al., 2018). These studies had markedly different controls including no contact (Ball et al., 2002), active control (Edwards et al., 2005; Corbett et al., 2015; Rizkalla, 2015), and unclear control (Chen et al., 2018). They also used different validated scoring systems such as the Timed IADL (Edwards et al., 2005) and the Minimum Data Set-Home Care (Ball et al., 2002; Corbett et al., 2015) to measure IADL ability.

### 3.2. Mild Cognitive Impairment

Five studies included participants with mild cognitive impairment. Studies were smaller than those involving normal cognition and involved a mean of 115 participants. Follow up also varied widely from 2.5 months (Guili et al., 2016) to 24 months in one cohort of one study (Eleni et al., 2017). All five of these studies were performed in an ambulatory clinic and also included a variety of group, individual, and computerized tasks.

The results of these studies are summarized in Table 4. Only one of the five studies reported improvement in measures of IADL (Eleni et al., 2017). This study had two cohorts with one receiving intervention for 12 months and another for 24 months. Their study showed greater improvement with longer intervention. Similar to the studies involving those with normal cognition, these studies had widely different controls including no contact (Belleville et al., 2018), no therapy (Eleni et al., 2017), and active control (Fiatarone Singh et al., 2014; Law et al., 2014; Guili et al., 2016). These studies also used different scoring systems for measures of IADL.

### 3.3. Cognition and Independence

Twelve of the thirteen studies reported on measures of cognition. Of these, eleven reported improvement using a variety of validated tests of cognition, such as the ADAS-Cog and the Hopkins Visual Learning Test. Only one (McDougall et al., 2010) did not find a significant change at follow up.

None of the studies reported showed a change in the ability to live independently.

## 4. Discussion

### 4.1. Normal Cognition

Overall, our review has found a number of studies that report an improvement in IADL function after cognitive training. The majority of the studies focused on those with normal cognition, suggesting that cognitive training may be of more benefit to this subgroup. These studies were quite heterogenous, with all using different protocols and assessment tools. Therefore, we are unable to comment on what type of protocol or assessment is most effective.

The maintenance of improved IADL function from cognitive training is also difficult to comment on. Most of the studies selected in our review had follow ups of between eight weeks to one year. A select few had longer follow ups up to two years. Only one study so far, the ACTIVE trial, has released ten year follow up data. In their analysis, they report maintenance of self reported IADL function up to ten years post intervention [26]. This is encouraging in that we may simply need longer follow up periods to show a significant change in daily functioning.

### 4.2. Mild Cognitive Impairment

We found only one study that met our inclusion criteria including participants with mild cognitive impairment that showed improvement in IADL function. This suggests that cognitive training may not be as effective in those with a diagnosis of mild cognitive impairment. Our hypothesis is that with mild cognitive impairment, there already exists a degree of difficulty with learning, processing, and planning. This could impact the ability to acquire skills from cognitive training. However, the caveat would be that we lack data regarding this subgroup, either from difficulty with recruitment or the fact that mild cognitive impairment encompasses a wide spectrum of presentations.

The study by Eleni et al. [25] also interestingly showed a greater improvement with longer therapy of 24 months versus 12 months. This further reinforces the notion that longer studies may be able to show a greater effect that what has been previously reported in the literature.

### 4.3. Cognition and Independence

Our findings also support what has already been reported in the literature, that cognitive training does have a role in improving measures of cognition. We are unable to make any conclusions, however, regarding its effect on the ability to live independently. This may again reflect the lack of long-term data, since changes in independence are more likely to occur over years rather than months.

### 4.4. Limitations

Our review has a number of limitations. First, the data regarding cognitive training is heterogenous. There is no standardized methodology for the screening of participants, type of experimental control, or a unified measure of outcome. The studies that we selected used widely different protocols and reported outcomes using almost completely different scales. Effect sizes were seldom reported. Previous reviews, such as by Mewborn et al. [11], elected to not include studies that did not report effect sizes when selecting studies. While this would make inferential statistical analysis possible, the paucity of trials that report IADL function would limit our review to a paltry few studies.

Study selection was also predominantly performed by one reviewer and therefore increases the risk of selection bias. This was mitigated with a second independent reviewer when uncertainty regarding the appropriateness of a study was present.

### 4.5. Future Directions

We have identified a large gap in our current knowledge of the efficacy of cognitive training. Many studies have focused on specific domains of cognition, but few studies so far have delved into its effect on global function and the maintenance of independence. Given multiple calls to action to improve the health of our elderly patients and the limited evidence-based interventions that are available, it would be vital to identify any new tool that can be widely adopted. Thus, larger and more robust studies that focus on more generalizable outcomes are needed.

Given our findings, it would be reasonable to support further long-term studies in cognitive training. Future investigations may be able to delineate whether or not there is a significant difference in the effect on those with normal cognition versus mild cognitive impairment. The effect on the ability to function independently with sustained training has also yet to be determined and would be an excellent topic of research.

## 5. Conclusions

The role of cognitive training and its effect on daily functioning in terms of instrumental activities of daily living remain unclear. While a significant effort has been made to show improvement in terms of cognition and its many domains, few studies have reported on its effect on function. It also remains to be seen whether or not functional gains are maintained in the long term. Our review does show promise that there is indeed a signal that supports cognitive training having a role in improved function. Further research in this area should be supported, with long term follow up being a crucial aspect that needs to be addressed.

## Figures and Tables

**Table 1:** Study characteristics involving randomized control trials (RCTs) with participants with normal cognition.

Authors	Year	Sample Size (n)	Average Age	Baseline MMSE	Country	Duration	Follow Up (Months)	Training Protocol
Ball K et al. [6]	2002	2832	73.6	27.3/30	US	10 sessions	24	Memory training, reasoning training or speed-of-processing training with classroom exercises or computer tasks
Edwards et al. [16]	2005	126	76	28.1/30	US	10 sessions	2.5	Speed of processing intervention
McDougall et al. [17]	2010	265	75	28/30	US	12 sessions	26	Memory training with classes on memory improvement
McDaniel et al. [18]	2014	96	65	29/30	US	24 sessions	6	Computerized and in-person simulations and activities
Lampit et al. [19]	2014	77	72.1	28/30	Australia	36 sessions	12	Computerized cognitive training
Corbett et al. [7]	2015	2912	65	NR	UK	10 min per day	6	Online reasoning and memory tasks
Rizkalla [20]	2015	60	72.5	18.8/21	US	20 sessions	4	Self-administered sessions of executive functioning, memory and emotion training modules
Giuli et al. [21]	2016	100	72.4	28/30	Italy	10 sessions	2.5	Training focusing on lifestyle changes and education
Chen et al. [22]	2018	86	68.6	NR	China	10 sessions	2.5	Memory and reasoning tasks, divided into low ecological with weak connection to daily activities and high ecological that simulate daily activities.

RCT = Randomized control trial, MMSE = Mini-Mental Status Examination, UK = United Kingdom, US = United States, NR = Not reported.

**Table 2:** Study characteristics involving RCTs with participants with mild cognitive impairment.

Authors	Year	Sample Size (n)	Average Age	Baseline MMSE	Country	Duration	Follow Up (Months)	Training Protocol
Fiatarone Singh et al. [23]	2014	100	70.1	27/30	US	48 sessions	18	Cognitive training vs. resistance training vs. combined
Law et al. [24]	2014	83	73.8	24/30	Hong Kong	13 sessions	6	Simulated functional tasks exercises
Giuli et al. [21]	2016	97	76.3	25.7/30	Italy	10 sessions	2.5	Training focusing on lifestyle changes and education
Eleni et al. [25]	2017	151	70.5	27.9/30	Greece	34 sessions	12 (74 pts) and 24 (41 pts)	Multi-component tasks with computer, paper and pencil, and musical stimuli components
Belleville et al. [8]	2018	145	72	NR	Canada	8 sessions	6	Memory and attentional control strategies vs. psychosocial intervention

RCT = Randomized control trial, MMSE = Mini-Mental Status Examination, UK = United Kingdom, US = United States, NR = Not reported.

**Table 3:** Study results with participants with normal cognition.

Authors	Protocol	Control	Measure of IADL	Conclusion	Measure of Cognition	Conclusion
Ball K et al.	Memory training, reasoning training or speed-of-processing training with classroom exercises or computer tasks	No-contact	Minimum Data Set-Home Care (MDS-HC)	Observed decline rates below established population norms.	Reasoning assessment and speed-of processing assessment	Improvement in Memory for the Memory Training Cohort, Reasoning in the Reasoning Training Cohort, Speed in the Speed Training Cohort
Edwards et al.	Speed of processing intervention	Computer-contact with Internet training	Timed IADL	Intervention group performed more quickly and accurately.	Useful Field of View (UFOV)	Significantly better performance
McDougall, et al.	Memory training with classes on memory improvement	Health Promotion Classes	Direct Assessment of Functional Status (DAFS)	No significant change.	Hopkins Verbal Learning Test Revised (HVLT-R)	Unchanged
McDaniel et al.	Computerized and in-person simulations and activities vs. aerobic exercise	Home-exercise and health education sessions	Simulated activities: Cooking Breakfast, Virtual Week, Memory for Health Information	Improvement in memory performance in Virtual Week, but not in Cooking Breakfast or Memory for Health Information.	Not Applicable	Not Applicable
Lampit et al.	Computerized cognitive training	Active control with National Geographic videos	Bayer ADL scale	No significant change.	Global Cognition Score	Significant effect that was sustained over 12 months
Corbett et al.	Online reasoning and memory tasks	Internet-based tasks	Minimum Data Set-Home Care (MDS-HC)	Significant benefit to IADLS in both Reasoning and General Cognitive Training Groups.	Baddeley Grammatical Reasoning, Hopkins Verbal Learning Test	Considerable generalizable impact
Rizkalla	Self-administered sessions of executive functioning, memory and emotion training modules	Active control with word searches, short stories and MCQs	Disability assessment for dementia (DAD) and Functional rating scale (FRS)	Small significant change in DAD and FRS post treatment.	Brief cognitive rating scale (BCRS)	Improvement in global cognition
Giuli et al.	Training focusing on lifestyle changes and education	General psychoeducational support	IADL/ADL Assessment	No significant change.	Battery of cognitive tests including: Forward and backward verbal span, Prose memory test	Improvement in forward verbal span in healthy aging, improvement in Prose memory test, word pairing in mild cognitive impairment (MCI)
Chen et al.	Memory and reasoning tasks, divided into low ecological with weak connection to daily activities and high ecological that simulate daily activities.	Not described	Observed Task of Daily Living (OTDL-C)	Improvement in everyday problem solving.	Spatial and numerical working memory, and reasoning	Improvement in memory for memory training group and improvement in reasoning in reasoning training group

**Table 4:** Study results with participants with mild cognitive impairment.

Authors	Protocol	Control	Measure of IADL	Conclusion	Measure of Cognition	Conclusion
Fiatarone Singh et al.	Cognitive training vs. resistance training vs. combined	Sham cognitive and resistance training	Bayer-ADL	No group effect	ADAS-Cog	No difference between intervention and sham
Law et al.	Simulated functional tasks exercises	Computer based cognitive training	Lawton IADL	Significant improvement in IADLs post intervention but not at follow-up.	Neurobehavioral Cognitive Status Examination (NCSE)	Improvement in multiple domains of cognition
Giuli et al.	Training focusing on lifestyle changes and education	General psychoeducational support	IADL/ADL Assessment	No significant change in experimental group.	Battery of cognitive tests including: Forward and backward verbal span, Prose memory test	Improvement in forward verbal span in healthy aging, improvement in Prose memory test, word pairing in MCI
Eleni P et al.	Multi-component tasks with computer, paper and pencil, and musical stimuli components	No therapy	Functional Cognitive Assessment Scale (FUCAS)	Better performance in daily activities at 12 months, with the 24 month cohort having better performance than the 12 month cohort.	Rey Auditory Verbal Learning test	Improved verbal learning ability and delayed verbal recall
Belleville et al.	Memory and attentional control strategies vs. psychosocial intervention	No contact	Complex activities of daily living (ADL-PI)	No improvement but did increase self-reported use of strategies in daily life.	Delayed Memory Composite Score	Improved and persisted over 6 months
